# Stable Isotope and Trace Element Studies on Gladiators and Contemporary Romans from Ephesus (Turkey, 2nd and 3rd Ct. AD) - Implications for Differences in Diet

**DOI:** 10.1371/journal.pone.0110489

**Published:** 2014-10-15

**Authors:** Sandra Lösch, Negahnaz Moghaddam, Karl Grossschmidt, Daniele U. Risser, Fabian Kanz

**Affiliations:** 1 Department of Physical Anthropology, Institute of Forensic Medicine, University of Bern, Bern, Switzerland; 2 Center of Anatomy and Cell Biology, Medical University of Vienna, Vienna, Austria; 3 Department of Forensic Medicine, Medical University of Vienna, Vienna, Austria; Ohio State University, United States of America

## Abstract

The gladiator cemetery discovered in Ephesus (Turkey) in 1993 dates to the 2^nd^ and 3^rd^ century AD. The aim of this study is to reconstruct diverse diet, social stratification, and migration of the inhabitants of Roman Ephesus and the distinct group of gladiators. Stable carbon, nitrogen, and sulphur isotope analysis were applied, and inorganic bone elements (strontium, calcium) were determined. In total, 53 individuals, including 22 gladiators, were analysed. All individuals consumed C_3_ plants like wheat and barley as staple food. A few individuals show indication of consumption of C_4_ plants. The δ^13^C values of one female from the gladiator cemetery and one gladiator differ from all other individuals. Their δ^34^S values indicate that they probably migrated from another geographical region or consumed different foods. The δ^15^N values are relatively low in comparison to other sites from Roman times. A probable cause for the depletion of ^15^N in Ephesus could be the frequent consumption of legumes. The Sr/Ca-ratios of the gladiators were significantly higher than the values of the contemporary Roman inhabitants. Since the Sr/Ca-ratio reflects the main Ca-supplier in the diet, the elevated values of the gladiators might suggest a frequent use of a plant ash beverage, as mentioned in ancient texts.

## Introduction

**Figure 1:**
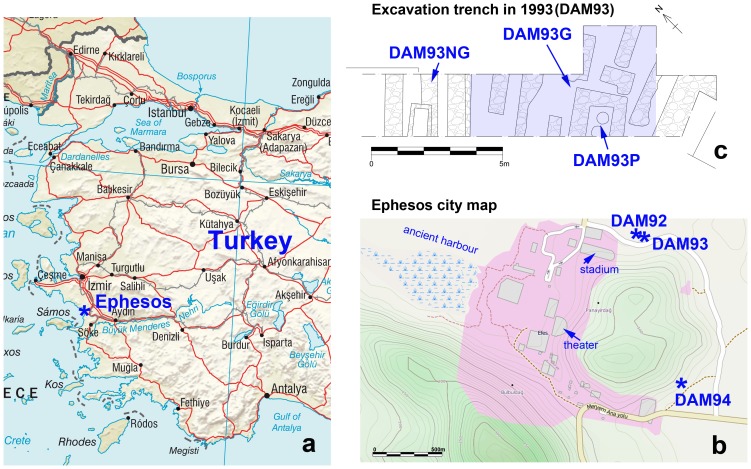
Study area at the west coast of Turkey. (a) Geographical location of ancient Ephesus. (b) City map of Roman Ephesus with investigated excavations sites. (c) Details of the excavation trench in 1993 (DAM93) including the gladiator cemetery (DAM93G). Images compiled with modified data from OpenStreetMap and CIA maps.

There are various archaeological publications about the unique cultural phenomenon of Roman gladiators [1], [2], [3], [4], [5], [6], [7], [8], [9] as well as artifacts associated with them [10], [11], [12], though the recovery of human remains from gladiators is extremely rare [13], [14]. Alleged arena fighters were found only at a few excavation sites: Pompeii in Italy [15], Eboracum (York, UK) [16], Augusta Treverorum (Trier, Germany) [17], and Colonia Augusta Aroe Patrensis (Patras, Greece) [18]. However, the verification of these findings remains uncertain. In 1993, a gladiator cemetery was discovered in Ephesus, Turkey [19] (Figure 1a). Most individuals from this site exhibited trauma which supports the assumption that they were gladiators [13], [14]. The trauma pattern confirms what written sources mention about the rules for gladiator fights.

Historical sources report that socially stratified Roman populations had diverse nutrition. Recently, several isotope analyses were conducted on human bones from Roman times, especially from Italy [20], [21], [22], [23], [24], Croatia [25], Britain [16], [26], [27], [28], Tunisia [29], and Egypt [30], [31], [32]. In contrast, little isotopic work has been done on skeletal series from the geographic region of Turkey [33], [34], [35], [36], [37]. Contemporary Roman texts mention that gladiators consumed a specific diet called “*gladiatoriam saginam*”, which included barley and bell beans (*vicia faba*) [38], [39]. Their consumption of barley led to the derogatory nickname “*hordearii*” (barley eaters) [40]. Presuming the historical reports on gladiator diet habits were accurate, it might be possible to detect differences in stable isotope and/or trace element ratios in bones of gladiators and contemporary “ordinary” Romans. Therefore, the aim of this study was to reconstruct possible diet restrictions of gladiators in comparison to contemporary inhabitants of Roman Ephesus. Stable isotopes (C, N, S) and the inorganic bone compounds (Sr, Ca) were investigated to obtain information about nutrition and social stratification.

### Stable isotope analysis

Stable light isotope ratio analysis of bone collagen is an established method in bioarchaeology. It reveals important information about nutrition, life history, and migration in past populations [41], [42], [43], [44], [45]. Bones and teeth are the most commonly preserved human tissues in the archaeological record. Collagen is the main protein in bone that provides the source for organic carbon (^13^C/^12^C), nitrogen (^15^N/^14^N) and to a lesser extent sulphur (^34^S/^32^S). Because bone is a living tissue that turns over regularly within the lifetime of an individual, isotope ratios reflect average values.

The analysis of stable carbon isotopes provides important information about plant consumption. Due to fractionation, the consumer's δ^13^C value is enriched by +5‰ when vegetable food is metabolised. Stable carbon isotope ratios in bone collagen are analysed to differentiate between individuals with higher intake of C_3_ (e.g. wheat, barley) and C_4_ (e.g. millet, maize) plants which differ in their photosynthetic pathway [41], [46], [47].

Stable nitrogen isotopes in bone collagen reflect the intake of animal protein, especially meat and dairy products. Like stable carbon isotope ratios, the δ^15^N values also give information about the trophic level. Due to fractionation the approximate shift is about +3‰ to +5‰ in bone collagen [43], [48]. In an ecosystem, the δ^15^N value increases towards the top of the food chain.

The analysis of the bone collagen sulphur isotope ratio as a multiple isotopic approach can shed light on additional aspects of ancient populations' diets [49]. The δ^34^S in bone collagen is passed along the food chain with a small fractionation of approximately −1‰ and directly reflects the δ^34^S values of the consumed food [27], [37]. Compared to freshwater and terrestrial environments, there is a very high concentration of compounds containing sulphur in the ocean [50]. δ^34^S values in freshwater and terrestrial ecosystems range from −22‰ to +22‰ [51], [52]. Terrestrial mammals have δ^34^S values lower than +10‰, whereas organisms in marine ecosystems have values around +20‰ [53]. The “sea spray effect” describes the high amount of marine sulphates in coastal regions and islands as a result of evaporation and rainfall [54]. Unlike carbon and nitrogen isotope analysis, δ^34^S in ancient bone collagen is addressed in only few publications [27], [51], [52], [53], [54], [55], [56], [57], [58]. Therefore, few comparative data are available for this study.

### Trace element analysis

Through analysis of the inorganic mineral of bone – mainly a bio-apatite similar to the more crystalline hydroxyapatite – it should be possible to identify the main Ca supplier in the diet [59] through its specific Sr/Ca-ratio which can be reconstructed from the Sr/Ca ratios found in bone.

Sr and Ca behave similarly in building up the hydroxyapatite in the mineral bone fraction due to their similar chemical properties. The pathways of Sr in biological systems and especially in the food web have been thoroughly investigated because of concerns regarding the biological effects of radioactive Sr-90 fallout caused by atmospheric nuclear tests in the 1950s and 1960s [60], [61], [62], [63], [64]. As a result, the Sr metabolism and the Sr distribution in various organisms are well known. It has been shown that Sr serves no known metabolic function. Compared to Ca, the uptake of Sr into the body underlies discriminating restrictions during its passage from the digestive tract into the bloodstream. Mammals absorb only 20–30% of the Sr intake, but once in the body, virtually the entire amount of Sr accumulates in bone and teeth as a substitute for Ca in the apatite lattice [60], [65], [66], [67], [68]. Due to the preferred uptake of Ca compared to Sr and the resulting “biopurification” of Ca in the food chain, it was long believed that Sr functions as a dietary plant-to-meat ratio indicator [67]. It was assumed that a high Sr amount in the bone reflected a high vegetarian intake, and a low Sr amount indicated a diet rich in meat. Therefore, studies of Sr/Ca-ratios for the reconstruction of diet were introduced in 1965 for extinct animals and in the 1970s and early 1980s for prehistoric humans [69], [70], [71], [72], [73], [74], [75]. Since then, numerous studies have dealt with this subject. Thus, we refer to detailed review articles(e. g. by [76], [77], [78], and [59]). Two major issues with this concept of diet reconstruction have to be highlighted:

First, the dietary plant-to-meat ratio approach experienced a major correction by Burton and Wright [79]. They showed that Sr/Ca ratios in bone reflect the Sr/Ca ratio of the strongest Ca supplier rather than the whole plant-to-meat ratio of a multicomponent diet. This limits future Sr/Ca studies to identifying the major Ca source of the diet.

A second major drawback for the investigation of trace elements arose from the fact that bones buried in soil undergo diagenetic alterations [59], [80]. Many attempts were made to identify and quantify diagenetic changes to Sr/Ca-ratio with the target to reconstruct original Sr/Ca-ratios (summarized in [59]). Because of the complexity and variability of soil processes, this seems impossible up to now. Therefore, the scientific focus has shifted from trace elements to stable isotopes for diet reconstruction in recent years. Only few combined studies on trace elements and stable isotopes are available. Though we believe that trace element and stable isotope investigations complement each other and have the potential to draw a more comprehensive picture of ancient diets.

### Gladiators

The social group of gladiators mainly consisted of prisoners of war, slaves, and condemned offenders. The Roman jurisdiction had two judgements that forced people to fight in the arena [8]. Contestants with swords (*ad gladium*) had to fight in the arena without prior special training, which in most cases meant certain death. Gladiator school participants (*ad ludum*) got appropriate training and education. They could prove their ability for social reintegration in the course of the fights. There were also “volunteer” gladiators regardless of the about 1∶9 probability of dying in a gladiator fight in the 1^st^ century AD [81]. Common citizens, senators, noblemen, and even emperors could pursue training in gladiator schools (*ludus*). Thus, the occupational group of gladiators probably consisted of males from almost all social groups of the Roman society [8], [81]. Therefore, occupational group here refers to participants of the gladiator school.

Gladiator fights as a socially institutionalized spectacle represent a unique phenomenon in human history. Ancient texts, ceramic artefacts, and iconography were subjected to scientific, archaeological, and historical investigation for a better understanding of gladiator fights and their cultural and social context in ancient Roman society [1], [2], [4], [5]. In recent years, additional data about the use of weaponry and protective gear were collected through experimental archaeological studies [9].

For Ephesus, gladiator fights are reported since 69 BC when they were held under the auspices of the Roman governor Lucullus [82]. Artefacts, mainly oil lamps and graffiti with fighter illustrations, suggest that gladiator fights became increasingly important over time and peaked out in the 2^nd^ and 3^rd^ century AD when Ephesus was the capital of the province Asia.

## Materials and Methods

### Ethics Statement

All necessary permits for the excavations, sampling, shipping and conducted analyses were obtained from the legal representative (Directorate-General for Antiquities and Museums of Ankara, Turkey).

### Sampled humans

Human remains from the gladiator cemetery (DAM93G) and from the three other excavation complexes marked as DAM92, DAM93NG, and DAM94 were examined (Fig. 1b). The excavations were conducted between 1992 and 1994. The complexes date to the 1st to 3rd century AD, the gladiator cemetery in particular dates to the 2nd to 3rd century AD [82]. Detailed information is given in the excavation reports [83], [84] and in the anthropological reports [85], [86], [87], [88]. The burial sites DAM93G and DAM93NG are located at the northern foot of the Panayırdağ hill about 300 m east of the ancient stadium, between a procession route and an ancient road (Fig. 1b). The human samples from DAM93NG derive from 8 female and 8 male individuals unearthed in 1993 in direct proximity to the gladiator cemetery. The excavation in the nearby “fig garden” revealed a sarcophagus filled with soil and remains of 3 females and 3 males (DAM92). At the opposite side of the Panayırdağ DAM94 was located and revealed burials of 7 females and 8 males in various sarcophagi.

**Figure 2:**
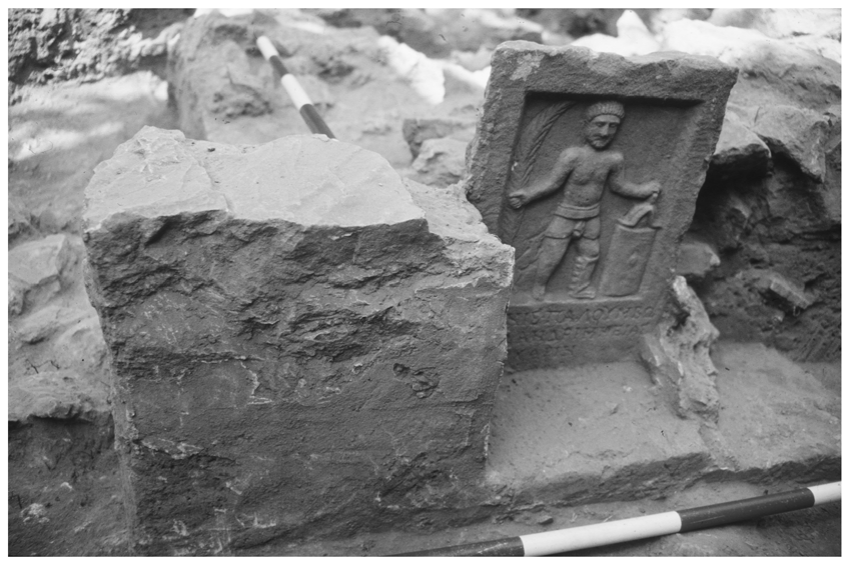
In situ gladiator tombstone excavated in the cemetery.

DAM93G (the gladiator cemetery) mainly consisted of graves with plain walls and fixed tombstones with illustrations of different types of gladiators (Fig. 2). The human remains were found in an approximately 3 m thick layer in a 20 m^2^ area (Fig. 1c). Commingled bones indicate that the site was used multiple times over an extended period [83]. Therefore, the documentation and recovery of human remains were carried out according to mass grave excavation methods [89], [90]. The morphologic-anthropological examination of the individuals' sex and age was performed as per [91], [92], [93], [94], [95] and [96]. 53 individuals were analyzed for stable isotope ratios and 35 individuals for Sr/Ca-ratios (Table 1).

**Table 1:** Individual data of stable carbon (C), nitrogen (N) and sulfur (S) isotope ratios for collagen and strontium (Sr), calcium (Ca) as well as Ca/P-ratios and Sr/Ca-ratios.

	Excavation complex	Sample-ID	Bone	Sex	δ^13^C [‰]_V-PDB_	δ^15^N [‰]_AIR_	δ^34^S [‰]_V-CDT_	%C_coll_	%N	%S	% Coll	C/N mol	C/S mol	N/S mol	Ca [mg/g]	Ca/P	Sr [μg/g]	Sr/Ca μg/mg]
Gladiators	DAM93G	EPH-DAM 128/93 rFEM 5	femur	M	−19.1	7.6	5.5	41.5	15.4	0.10	2.3	3.1	2.8	0.6	357.2	2.19	454.2	1.27
	DAM93G	EPH-DAM 146/93 rFEM 1	femur	M	−19.3	10.6	7.5	39.3	14.7	0.06	2.1	3.1	2.0	0.4	362.5	2.25	583.1	1.61
	DAM93G	EPH-DAM 155/93 rFEM 2	femur	M	−19.0	9.2	8.4	35.9	14.0	0.10	1.8	3.0	1.7	0.4	365.2	2.21	380.5	1.04
	DAM93G	EPH-DAM 155/93 rFEM 3	femur	M	−18.9	9.8	8.3	40.6	14.9	0.07	2.6	3.2	1.8	0.4	358.3	2.18	429.4	1.20
	DAM93G	EPH-DAM 155/93 rFEM 4	femur	M	−19.4	8.7	8.0	38.2	15.1	0.12	2.2	3.0	1.9	0.4	358.8	2.16	279.4	0.78
	DAM93G	EPH-DAM 155/93 rFEM 5	femur	M	−19.0	8.4	8.9	40.6	15.4	0.11	2.1	3.1	1.7	0.3	383.7	2.33	562.8	1.47
	DAM93G	EPH-DAM 155/93 rFEM 6	femur	M	−18.8	8.7	8.2	41.1	16.3	0.11	5.0	2.9	2.0	0.4	349.1	2.23	335.3	0.96
	DAM93G	EPH-DAM 187/93 rFEM 2	femur	M	−18.5	9.4	9.7	42.8	15.9	0.10	5.2	3.1	1.6	0.3	367.7	2.22	630.5	1.71
	DAM93G	EPH-DAM 187/93 rFEM 4	femur	M	−19.0	9.6	5.7	41.7	16.4	0.10	4.5	3.0	2.9	0.5	341.6	2.26	449.0	1.31
	DAM93G	EPH-DAM 187/93 rFEM 7	femur	M	−19.3	9.4	9.0	38.2	15.4	0.10	3.1	2.9	1.7	0.3	358.7	2.24	434.9	1.21
	*DAM93G*	*EPH-DAM 187/93 rFEM 10*	*femur*	*M*	−*19.0*	*7.6*	*8.1*	*39.9*	*17.2*	*0.15*	*1.9*	*2.7*	*2.1*	*0.3*	374.5	2.25	543.0	1.45
	DAM93G	EPH-DAM 187/93 rFEM 6	femur	M	−17.8	8.9	5.9	41.7	15.6	0.15	1.1	3.1	2.6	0.5	355.8	2.29	379.6	1.07
	*DAM93G*	*EPH-DAM 199/93 rFEM 1*	*femur*	*M*	−*19.1*	*10.7*	*7.9*	*27.8*	*13.2*	*0.05*	*4.6*	*2.5*	*1.7*	*0.3*	374.1	2.35	724.9	1.94
	DAM93G	EPH-DAM 199/93 rFEM 3	femur	M	−18.6	9.7	6.4	40.2	15.4	0.12	2.2	3.0	2.4	0.5	370.1	2.33	712.9	1.93
	DAM93G	EPH-DAM 199/93 rFEM 4	femur	N/A	−18.8	9.2	8.9	40.5	15.4	0.08	3.5	3.1	1.7	0.3	N/A	N/A	N/A	N/A
	DAM93G	EPH-DAM 248/93 rFEM 1	femur	M	−19.2	8.6	4.5	45.3	17.0	0.10	2.4	3.1	3.8	0.7	361.6	2.26	499.0	1.38
	DAM93G	EPH-DAM 248/93 rFEM 3	femur	M	−19.3	10.1	8.7	41.3	15.5	0.11	3.8	3.1	1.8	0.4	376.9	2.27	359.4	0.95
	DAM93G	EPH-DAM 248/93 rFEM 5	femur	M	−19.1	8.8	8.8	41.6	15.4	0.12	2.8	3.2	1.7	0.4	355.3	2.26	405.7	1.14
	DAM93G	EPH-DAM 248/93 rFEM 6	femur	M	−18.2	9.9	11.2	42.7	16.7	0.10	4.2	3.0	1.5	0.3	359.2	2.20	453.5	1.26
	DAM93G	EPH-DAM 76/93 rFEM 2	femur	M	−18.5	11.2	7.0	42.6	15.9	0.11	3.9	3.1	2.3	0.4	365.0	2.29	334.5	0.92
	DAM93G	EPH-DAM 78/93 rFEM 3	femur	M	−18.4	9.2	6.9	43.6	16.2	0.11	3.9	3.1	2.4	0.5	373.0	2.27	376.4	1.01
	DAM93G	EPH-DAM 83/93 rFEM 2	femur	M	−19.4	9.8	3.4	39.4	15.1	0.09	4.4	3.0	4.4	0.9	360.7	2.20	330.7	0.92
NG-males	DAM93NG	EPH-DAM 106/93 FEM	femur	M	−19.1	8.8	6.4	40.9	15.4	0.15	2.5	3.1	2.4	0.5	353.1	2.26	248.7	0.70
	DAM93NG	EPH-DAM 108/93 MAN	mandibula	M							<LOD				N/A	N/A	N/A	N/A
	DAM94	EPH-DAM 143/94 lFEM 1	femur	M	−18.9	9.4	8.4	38.7	14.3	0.06	2.1	3.2	1.7	0.4	374.5	2.17	264.0	0.70
	DAM94	EPH-DAM 143/94 lFEM 2	femur	M	−19.0	10.4	7.6	37.8	14.6	0.08	2.5	3.0	1.9	0.4	361.8	2.24	301.4	0.83
	DAM94	EPH-DAM 163/94 lHUM 1	humerus	M	−18.8	8.8	5.7	41.7	16.0	0.10	3.6	3.0	2.8	0.5	370.9	2.25	226.4	0.61
	DAM94	EPH-DAM 163/94 lHUM 4	humerus	M	−18.8	8.9	6.8	38.5	15.6	0.06	2.3	2.9	2.3	0.4	368.9	2.26	269.2	0.73
	DAM94	EPH-DAM 163/94 lHUM 5	humerus	M	−19.6	10.5	7.0	41.2	16.2	0.06	2.4	3.0	2.3	0.4	359.3	2.29	302.9	0.84
	DAM93NG	EPH-DAM 191/93 rHUM	humerus	M	−19.0	8.8	8.8	38.9	14.9	0.09	3.0	3.0	1.7	0.3	356.5	2.25	195.7	0.55
	DAM94	EPH-DAM 191/94 lFEM 1	femur	M	−18.9	10.0	6.8	41.2	15.1	0.13	1.8	3.2	2.2	0.5	374.6	2.30	195.6	0.52
	DAM94	EPH-DAM 191/94 lFEM 2	femur	M	−18.9	9.0	5.4	37.5	14.9	0.06	2.4	2.9	2.8	0.5	358.0	2.25	236.7	0.66
	DAM94	EPH-DAM 204/94 lFEM-1	femur	M							<LOD				N/A	N/A	N/A	N/A
	DAM94	EPH-DAM 325+327/94 lHUM	humerus	M	−19.3	9.2	9.1	40.6	15.0	0.10	3.4	3.2	1.7	0.3	369.8	2.32	226.6	0.61
	DAM92	EPH-DAM FEIGEN 1/92 FEM 2	femur	M	−18.7	9.2	7.7	41.9	15.8	0.12	6.0	3.1	2.1	0.4	349.7	2.25	236.2	0.68
	DAM92	EPH-DAM FEIGEN 3/92 lPAT 8	patella	M	−18.9	9.1	6.9	42.3	15.5	0.11	5.1	3.2	2.2	0.5	N/A	N/A	N/A	N/A
	DAM92	EPH-DAM FEIGEN 3/92 TIB 4	tibia	M	−18.7	9.4	8.1	43.1	16.3	0.10	6.4	3.1	2.0	0.4	N/A	N/A	N/A	N/A
Females	DAM93NG	EPH-DAM 106/93 PAR 2	skull	F	−19.4	7.4	6.9	43.9	16.3	0.07	1.4	3.1	2.4	0.5	N/A	N/A	N/A	N/A
	*DAM94*	*EPH-DAM 174/94 rHUM 2*	*humerus*	*F*	−*19.0*	*8.3*	*5.8*	*31.6*	*14.4*	*0.07*	*2.2*	*2.6*	*2.5*	*0.4*	367.9	2.21	247.4	0.67
	*DAM93NG*	*EPH-DAM 214/93 MAN 2*	*mandibula*	*F*	−*19.7*	*4.8*	*4.8*	*25.2*	*13.4*	*0.06*	*1.7*	*2.2*	*2.8*	*0.5*	N/A	N/A	N/A	N/A
	DAM94	EPH-DAM 307/94 lHUM	humerus	F	−19.2	8.8	3.9	40.6	15.7	0.09	8.9	3.0	4.0	0.8	371.8	2.28	240.4	0.65
	DAM93NG	EPH-DAM 72/93 rFEM 1	femur	F	−18.2	9.5	11.2	38.7	14.8	0.06	3.3	3.0	1.3	0.3	370.7	2.26	236.0	0.64
	DAM92	EPH-DAM FEIGEN 2/92 MAN 1	mandibua	F							<LOD				N/A	N/A	N/A	N/A
	*DAM92*	*EPH-DAM FEIGEN 3/92 CLA 5*	*clavicula*	*F*	−*18.7*	*10.1*	*6.0*	*37.4*	*13.6*	*0.17*	*0.9*	*3.2*	*2.3*	*0.5*	N/A	N/A	N/A	N/A
	DAM92	EPH-DAM FEIGEN 3/92 lPAT 9	patella	F	−19.0	9.8	6.8	39.6	14.5	0.11	3.8	3.2	2.1	0.5	N/A	N/A	N/A	N/A
Infants	DAM94	EPH-DAM 127/94 rULN	ulna	N/A	−18.9	9.5	8.0	42.3	15.6	0.08	5.4	3.2	2.0	0.4	N/A	N/A	N/A	N/A
	DAM94	EPH-DAM 148/94 lPUB 1	pubis	N/A	−18.9	8.2	5.1	43.0	16.5	0.06	6.9	3.0	3.2	0.6	N/A	N/A	N/A	N/A
	DAM94	EPH-DAM 148/94 lPUB 2	pubis	N/A	−19.3	9.3	7.7	41.2	15.6	0.11	4.5	3.1	2.0	0.4	N/A	N/A	N/A	N/A
	*DAM94*	*EPH-DAM 174/94 lHUM 1*	*humerus*	*N/A*	−*19.1*	*7.8*	*4.7*	*36.5*	*15.1*	*0.08*	*5.0*	*2.8*	*3.2*	*0.6*	N/A	N/A	N/A	N/A
	DAM93NG	EPH-DAM 214/93 MAN 1	mandibula	N/A							<LOD				N/A	N/A	N/A	N/A
	DAM92	EPH-DAM FEIGEN 3/92 rFEM 3	femur	N/A							<LOD				N/A	N/A	N/A	N/A
	*DAM92*	*EPH-DAM FEIGEN 3/92 MAN 7*	*mandibula*	*N/A*							*0.3*				N/A	N/A	N/A	N/A
	*DAM92*	*EPH-DAM FEIGEN 3/92 MAN 6*	*mandibula*	*N/A*	−*19.14*	*8.61*	*5.64*	*33.9*	*11.7*	*0.16*	*0.7*	*3.4*	*2.1*	*0.6*	N/A	N/A	N/A	N/A

Individuals excluded due to collagen quality criteria are shown in *italic* type; LOD, limit of detection; N/A, not applied; F, female; M, male.

The sample ID (e.g. EPH-DAM 155/93 rFEM 2), consists of EPH for the location Ephesus, DAM for the excavation site Damianosstoa, 155 is the storage box number, 93 means the excavation year 1993 and rFEM-2 stands for the second right femur in this storage box. Retaining subsamples are stored at the Department of Forensic Medicine in Vienna. The remaining bones are labelled and saved in storage boxes in the sealed depot of the excavation house of the Austrian Archaeological Institute in Ephesus (Atatürk Mah., 1064 Sok., No 13, TR-35920 Selçuk/İzmir, Turkey).

### Sample for trace element diagenesis control

For investigation of diagenetic alterations of Sr/Ca-ratios, additional samples of cremated human bones and soils were taken. About 18 kg of cremated human bones from two large Pithoi (DAM93P) were available for investigations. Both of these ceramic containers, Pithos I (EPH-DAM-P-I/93) and Pithos II (EPH-DAM-P-II/93), were found in close proximity to the gladiator cemetery (Fig. 1c). Anthropological investigations revealed that at least 3 females and 13 male were cremated, and the remaining bone and teeth fragments were subsequently filled into the two Pithoi [87], [97]. A subsample of about 200 g of femur fragments from each Pithos was taken and homogenized to get a representative and average sample for all buried individuals.

Soil samples were retrieved from all four excavation locations (DAM92, DAM93G, DAM93NG, DAM94). Since during the initial excavations no chemical studies were planned, systematic sampling, such as taking complete soil columns, was not carried out. Retrospectively, it was possible to get soil from the inner lumen of the bones. Specimen identifiers were given according to the system used for the human remains with the ending SOIL (e.g. in EPH-DAM92-SOIL) (Table 2).

**Table 2:** Data of calcium (Ca); Ca/P-ratios, strontium (Sr), and Sr/Ca-ratios, as well as lanthanum (La) and pH for the analyzed samples of animal and cremated human bones respectively total soil and soil eluents.

Sample-ID	Specimen	Ca [mg/g]	Ca/P	Sr [μg/g]	Sr/Ca [μg/mg]	La [μg/g]	pH
EPH-DAM-P-I/93-HOMO	cremated human bone	368.1	2.18	211.9	0.58	N/A	N/A
EPH-DAM-P-II/93-HOMO	cremated human bone	376.2	2.17	229.8	0.61	N/A	N/A
EPH-DAM92-SOIL	soil	114.6	11.1	171.5	1.50	53.5	N/A
EPH-DAM93G-SOIL	soil	56.9	17.7	122.4	2.15	55.7	N/A
EPH-DAM93NG-SOIL	soil	42.2	31.2	102.7	2.43	50.0	N/A
EPH-DAM94-SOIL	soil	122.0	47.1	196.5	1.61	46.9	N/A
EPH-DAM92-ELU	soil eluent	868.9*	14.0	2.92*	3.36	<LOD	7.30
EPH-DAM93G-ELU	soil eluent	535.8*	19.3	1.52*	2.84	<LOD	7.72
EPH-DAM93NG-ELU	soil eluent	479.3*	24.4	1.46*	3.05	<LOD	7.66
EPH-DAM94-ELU	soil eluent	875.4*	52.0	2.78*	3.18	<LOD	7.14

*… μg/L.

### Collagen extraction and stable isotope ratios measurement

All samples were cleaned with distilled water and ground to bone powder. The collagen extraction followed the [98] and [41] procedure. 500 mg samples were treated with 1 M HCl to dissolve the bone mineral and then rinsed until neutral. To remove humic and fulvic acids, the samples were treated with 0.125 NaOH and gelatinized in warm 0.001 M HCl at 90°C for 17 h. Then, the samples were filtered and freeze-dried. The lyophilized collagen was weighed three times in tin capsules. Stable isotope ratios of carbon (^13^C/^12^C), nitrogen (^15^N/^14^N), and sulphur (^34^S/^32^S) were analysed by isotope ratio mass spectrometry (IRMS at Isolab GmbH). The mean value of all three measurements was calculated, and an internal standard was used to determine the analytic error. The data are presented in δ-notation in per mil (‰) relative to international defined standards for carbon (Vienna Pee Dee Belemnite, VPDB), nitrogen (Ambient Inhalable Reservoir, AIR), and sulphur (Canyon Diablo Troilit, CDT). The analytical error amounted to ± 0.1‰ for δ^13^C, ± 0.2‰ for δ^15^N and ± 0.3‰ for δ^34^S.

#### Collagen quality control

The collagen quality was verified as per [99], [41] and [100]. Collagen extracts with less than 1% collagen in proportion to their dry weight and data, with a molar C/N relation outside a 2.9–3.6 range were not taken into consideration. %C and %N values (43% and 15–16%) that strongly deviate from recent collagen values were also not taken into consideration.

### Trace element sample preparation and measurements

The bone sample preparation followed a protocol developed for spectroscopic trace element analysis in bone or teeth [101], [102], [103]. First, the bones were superficially cleaned with tap water and dried at room temperature. Available bones were sampled with a diamond-coated trepanation drill. Pieces of compact bone were cleaned with distilled water and degreased with diethylether. To remove possible diagenetic contaminations, specimens were etched for 4 min with concentrated formic acid and then washed with distilled water in an ultrasonic bath following the decontamination protocol by [104], respectively [105]. After drying and homogenisation in agate mortar, an aliquot of approximately 500 mg bone powder was ashed at 500°C for 12 h and wet-digested under pressure for 6 h at 110°C with 1.5 ml concentrated nitric acid in a Teflon bomb.

Additionally, bone drilling wastes were collected for each group and homogenised aliquots were checked for the presence of Lanthanum (La). It turned out that for all drill waste samples the concentration of La was below the limit of detection (0.09 µg/g). Therefore, 100 µg La were added as internal standard to the resulting digestions of all bone samples and filled up to a final volume of 20 ml with bi-distilled H_2_O. After a final dilution (1∶100), strontium, calcium and phosphorus were measured in triplicates by ICP-OES (Perkin Elmer Optima 3000XL) under optimized conditions (see below and [103]).

#### Soil sample preparation

For the soil pH determinations, 50 g of dry soil were placed in a conical flask (250 mL) and 100 ml of bi-distilled water was added and the flask was shaken for 2 minutes. The resulting suspension was allowed to set for about one hour until the pH electrode in the supernatant showed stable values (DIN ISO 10390) which were recorded. Subsequently, the supernatant was decanted and measured by ICP-OES to investigate soil leaching abilities occurring during the presence of rain water.

For the total digestion of soil samples, lithium metaborate fusion according to [106] was applied. 100 mg of dry soil was mixed with 500 mg lithium metaborate (LiBO_2_), transferred to a pre-ignited high-purity graphite crucible, and placed in the muffle furnace at 950°C for 15 min. The molten material was subsequently transferred into a Teflon beaker filled with 50 ml of 1N nitric acid and stirred until solution was completed. The solution was transferred to a volume flask and filled up to 100 ml with bi-distilled water and measured by ICP-OES.

#### Instrumentation characteristics for ICP-OES measurements

Spectral lines free of interferences were selected. The linear working ranges were investigated to determine the necessary dilutions for the measurements. Limits of detection (LOD) for each element were calculated by tripling the standard derivation of 11 measurements of the blank divided by the slope of the corresponding calibration curve. International reference standards SMR1486 (bone meal) and SMR2711 (Montana soil) were analyzed to monitor precision and accuracy of the sample pretreatment and ICP-OES measurements. The selected spectrometer wavelengths, necessary dilutions and the LODs for the bone samples, as well as the precisions and accuracies for bone and soil samples are given in Table 3.

**Table 3:** Instrumentation characteristics for the ICP-OES measurements.

Element	Ca	P	Sr	La
Wavelenght [nm]	317.933	213.618	407.771	379.478
Dilution	500 mg in 1 L	500 mg in 1 L	500 mg in 1 L	500 mg in 10 mL
LOD [μg/g]	44.8	145	0.282	0.0910
Recovery rate SMR1468 [%]	100.7	99.9	100.4	N/A
Precision* SRM1468 [%]	2.3	2.5	1.0	N/A
Recovery rate SMR2711 [%]	101.4	98.9	101.4	97.0
Precision* SRM1468 [%]	2.7	2.2	1.7	2.9

* … Precision was estimated from the deviation of six independent replicates.

LOD … Limit of detection if sample weight is 500 mg ashed (500°C) bone.

Since the accuracy turned out be lower than 2% for Ca, P and Sr in bone and soil, and the precision for determination of all elements was less than 3%, significant variation of actual differences in bone concentrations can be identified if they are greater than 3%.

### Statistical tests

Due to the sample size, the non-parametric Kruskal-Wallis test (3 parameters) and Mann-Whitney-U test (2 parameters) for independent samples were performed (SPSS Statistics 20). The aim was to detect evidence against the null hypothesis that there is no difference between the gladiators and the contemporary Romans.

## Results

### Bone samples

Bone samples of 22 males from the gladiator cemetery (DAM93G) were analysed (Table 1). After the extraction, two samples were excluded due to insufficient collagen quality [41], [99], [107], [108]. Additionally, samples of 31 non-gladiator (NG) individuals (15 males, 8 females, 8 infants) from the complexes DAM92, DAM93NG, DAM94 were analysed, and had to be reduced to 20 samples (13 males, 4 females, 3 infants) due to quality control or insufficient collagen detection. Until now, hardly any collagen quality criteria for sulphur isotope analysis have been published [109]. All δ^34^S values were evaluated for this study except those from the individuals that were excluded due to the aforementioned collagen quality control criteria.

For the Sr/Ca bone mineral analysis, only the mid-shaft compacta of adult individuals' femora and humeri were sampled since the turnover rates of the diaphyseal regions of these long bones are similar and therefore comparable [103]. This reduced the initial sample size for the gladiators to 21 males and for the non-gladiators (NG) to 11 males and 3 females.

The gladiator samples show mean values of −18.9±0.4‰ for δ^13^C, 9.3±0.8‰ for δ^15^N and 7.5±1.9‰ for δ^34^S. The mean values of the NG- male samples are −19.0±0.2‰ for δ^13^C, 9.4±0.6‰ for δ^15^N and 7.3±1.1‰ for δ^34^S. In the female samples, the mean values are −18.9±0.5‰ for δ^13^C, 8.9±1.1‰ for δ^15^N and 7.2±3.0‰ for δ^34^S, whereas the infant samples show mean values of −19.0±0.2‰ for δ^13^C, 9.0±0.7‰ for δ^15^N and 6.9±1.6‰ for δ^34^S (Table 4).

**Table 4:** Basic statistics for stable isotope ratios and Sr/Cr data.

	δ^13^C [‰]_V-PDB_						δ^15^N [‰]_AIR_					δ^34^S [‰]_V-CDT_					Sr/Ca [μg/mg]					
Group	*n*	Mean	SD	Median	Min	Max	Mean	SD	Median	Min	Max	Mean	SD	Median	Min	Max	*n*	Mean	SD	Median	Min	Max
Gladiators	20	−18.9	0.4	−19.0	−19.4	−17.8	9.3	0.8	9.3	7.6	11.2	7.5	1.9	8.1	3.4	11.2	21	1.26	0.33	1.21	0.78	1.94
NG-males	13	−19.0	0.2	−18.9	−19.6	−18.7	9.4	0.6	9.2	8.8	10.5	7.3	1.1	7.0	5.4	9.1	11	0.68	0.10	0.68	0.52	0.84
Females	4	−18.9	0.5	−19.1	−19.4	−18.2	8.9	1.1	9.2	7.4	9.8	7.2	3.0	6.8	3.9	11.2	3	0.65	0.02	0.65	0.64	0.67
Infants	3	−19.0	0.2	−18.9	−19.3	−18.9	9.0	0.7	9.3	8.2	9.5	6.9	1.6	7.7	5.1	8.0	N/A	N/A	N/A	N/A	N/A	N/A
Total	40	−18.9	0.4	−19.0	−19.6	−17.8	9.3	0.8	9.2	7.4	11.2	7.4	1.7	7.6	3.4	11.2	35	1.03	0.39	0.95	0.52	1.94

NG, non gladiator; SD, standard deviation; N/A, not applied.

The non-parametric Kruskal-Wallis test for unpaired groups on the gladiators and the contemporary males and females shows no significant difference in the mean δ^13^C (asymptotic p = 0.828), δ^15^N (asymptotic p = 0.850) and δ^34^S (asymptotic p = 0.668) values for the contemporary individuals. Therefore, all NG adults (male and female) were subsequently tested against the gladiators by the non-parametric Mann-Whitney-U test for unpaired groups (Table 5). This revealed no significant differences (asymptotic p for δ^13^C = 0.986, δ^15^N = 0.957, δ^34^S = 0.478).

**Table 5:** Kruskal-Wallis respectively Mann-Whitney U tests performed for gladiator and non gladiator groups.

Group	Variables	*p*
Gladiators **vs** NG-males **vs** females	δ^13^C	0.828
	δ^15^N	0.850
	δ^34^S	0.668
	Sr/Ca	0.586
Gladiators **vs** NG-all (NG-males and females)	δ^13^C	0.821
	δ^15^N	0.775
	δ^34^S	0.407
	Sr/Ca	**<0.001**

Significant *p* values are bold; NG, non gladiator.

The gladiators' Sr/Ca data show a mean value of 1.26±0.33 µg/mg. The NG male samples have a mean value of 0.68±0.10 µg/mg and the females of 0.65±0.02 µg/mg. Samples of infants were not analysed for Sr/Ca.

The non-parametric Mann-Whitney-U test for unpaired groups on the (male) gladiators and the NG males and females reveals in the first step that there is no significant difference between the mean Sr/Ca (p = 0.586) for the NG male and female sub-samples. Therefore, the NG males and females are pooled (0.67±0.09 µg/mg) to increase the sample size and then tested against the gladiator group. This reveals a highly significant difference between the Sr/Ca-ratio means (p<0.001) of the gladiators and the NG inhabitants (Table 5).

### Cremated human bone and soil samples

Results for the other analyzed samples are given in Table 2. Resulting ratios for Ca, Sr respectively Sr/Ca for mean bone, total soil and soil eluent gradients are given in Table 6.

**Table 6:** Ratios of Ca and Sr for bone vs. total soil vs. soil eluent and bone vs. soil eluent gradients, as well as Sr/Ca-ratios in bone, soil and eluent for all investigated excavation locations.

	Ca-ratios		Sr-ratios		Sr/Ca-ratios [μg/mg]		
Location	bone/soil/eluent	bone/eluent	bone/soil/eluent	bone/eluent	bone	soil	eluent
DAM92	3.05: 1: 0.0076	402.5: 1	1.38: 1: 0.0170	81.0: 1	0.68	1.50	3.36
DAM93G	6.39: 1: 0.0094	678.0: 1	3.76: 1: 0.0124	302.3: 1	1.26	2.15	2.84
DAM93NG	8.53: 1: 0.0114	751.2: 1	2.21: 1: 0.0142	155.3: 1	0.63	2.43	3.05
DAM94	3.01: 1: 0.0072	420.1: 1	1.28: 1: 0.0142	90.2: 1	0.68	1.61	3.18

The bone samples from the cremations out of Pithos I and II showed Sr/Ca-ratios of 0.58 µg/mg respectively 0.61 µg/mg, which are comparable to the Sr/Ca-ratios found in bones of the non-gladiator group (0.67±0.09 µg/mg).

Differences in the element content of the total soil samples were found. Specifically, Ca in the samples from the DAM93 location was only about half of what it was at the other two locations. The Sr concentrations in the DAM93 soils were similar (DAM93G = 122.4 µg/g and DAM93NG = 102.7 µg/g), but significantly lower than the concentrations in the soils of DAM92 (171.5 µg/g) and DAM94 (196.5 µg/g). Both of these findings together result in higher Sr/Ca-ratios for the soil of the DAM93 (2.15 µg/mg and 2.43 µg/mg) location compared to DAM92 (1.50 µg/mg) and DAM94 (1.61 µg/mg). For all four soils a heterogeneous distribution of P was evident by Ca/P ratios varying from 11.1 to 47.1. Finally, La was present in all soil samples at similar concentrations of about 50 µg/g.

The bi-distilled water elution of the soil samples revealed low and similar alkaline pH values for all four soils, ranging from 7.14 to 7.72. Nevertheless, the pH values for the DAM93 soils (pH 7.72 and pH 7.66) turned out to be slightly higher than for the DAM92 soil (pH 7.30) and DAM94 soil (pH 7.14). In the eluent, Ca concentrations of 535.8 µg/L for DAM93G, 479.3 µg/L for DAM93NG, respectively 868.9 µg/L for DAM92 and 875.4 µg/L for DAM94, were found.

The results reveal that about 1% of the Ca content of the DAM93 soils (DAM93G = 0.94%; DAM93NG = 1.14%) was eluted, whereas only 0.76% (DAM92) respectively 0.72% (DAM94) was depleted from the two other soils (Table 6).

The Sr concentrations in the eluent of the soils were 1.52 µg/L for DAM93G and 1.46 µg/L for DAM93NG, 2.92 µg/L for DAM92 and 2.78 µg/L for DAM94. These values indicate that the relative amount of Sr depleted was similar and on average approximately 1.5% of the total soil content. In other words, about the same relative amount of Sr can be depleted from all soils, but the relative depletion rate is slightly higher than the depletion rates for Ca. Especially the different depletion of Ca from the soils produces higher and finally more homogenous Sr/Ca-ratios in the eluent, as they were evident in the total soil. Specifically, in the eluent, an average Sr/Ca-ratio of 3.11±0.22 µg/mg, ranging from 2.84 µg/mg to 3.36 µg/mg was observed, whereas in the total soil the average Sr/Ca-ratio was 1.92±0.44 µg/mg (1.50 µg/mg to 2.43 µg/mg).

For the ratios of total soil versus bone, respectively, soil versus eluent and the direct ratios of eluent versus bone, it was found that the concentrations of Ca were at least 3 times higher in the bones than in the soils, and for DAM93NG even 8.53 times higher. The Sr concentrations in bone were higher than in the surrounding soils for all four locations, but the gradients were lower than for Ca, about 3 times for the DAM93 locations and a bit less than 1.5 times for DAM92 and DAM94.

The bone to eluent ratios are the most interesting ones, since they reflect similar ratios as those that would have occurred when (rain-)water was present to immerse the buried bone for a certain time. About 400 times more Ca was found in the bones of DAM92 and DAM94, and about 700 times higher Ca concentrations in the bones from the DAM93 locations. The Sr ratios turned out to be lower than the Ca ratios again, about 100 times for the DAM92 and DAM94 locations and 150 times for DAM93NG, respectively 300 times for DAM93G. It is obvious that, although the absolute Sr concentration for both DAM93 elutions is more or less the same (1.52 µg/L and 1.46 µg/L), the differences in the bone-eluent ratios (approx. 300 respectively 150) arise from significant differences in the Sr concentrations of the bones.

## Discussion

### Stable isotopes

The different groups' stable isotope data (gladiators, NG males/females) do not differ significantly. The sulphur isotope ratios show the greatest and the carbon ratios the lowest standard deviation.

### Carbon

All individuals consumed C_3_ plants like wheat and barley as staple food. Pliny the Elder reported in his *Naturalis historia* that barley (*hordearii*), a C_3_ plant, was a main component of the gladiators nutrition which matches our findings [9]. However, most NG individuals apparently also had C_3_ plants as a staple food since no statistical difference is detectable. A few individuals show more positive δ^13^C values. The most probable cause for this is an increased consumption of C_4_ plants such as millet. Millet was an important nutrition crop in Eurasia and its consumption has already been verified in several ancient societies by stable isotope investigations [110], [111], [112], especially since the Middle Bronze Age in Italy [113]. [21] published similar δ^13^C data for imperial Rome Isola Sacra (−18.8‰ versus −18.9‰ here). They mention ancient literature in which millet is described as an animal food and as less desirable for human consumption under “normal” circumstances. In their study, the δ^13^C values of the faunal remains are more negative than those of the humans, so it was assumed that the terrestrial herbivores basically fed on C_3_ plants. Although animal bones have not been investigated yet for reference in our study, we agree with [21]. If the δ^13^C values were caused by the consumption of millet-fed animals, then higher δ^15^N values would be expected for the Ephesus population.

**Figure 3:**
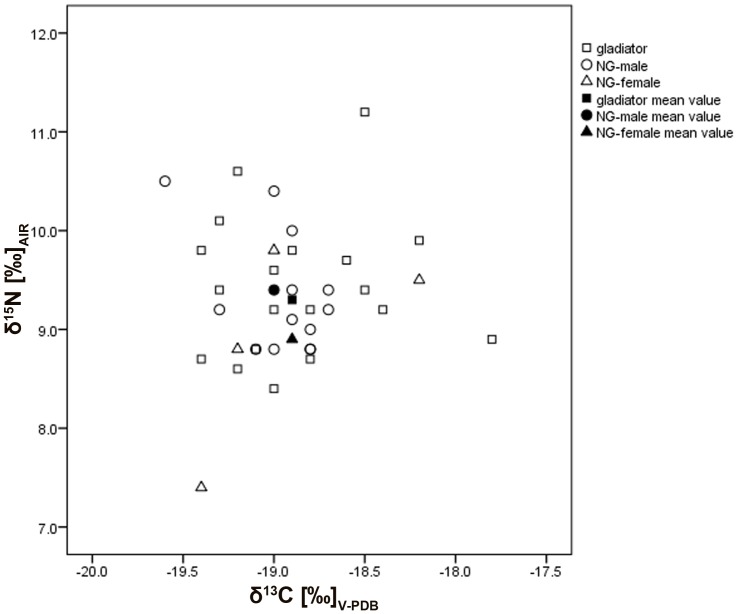
Stable δ^13^C and δ^15^N ratios. Comparison between different groups (gladiators, NG-males, and NG-females).

One of the individuals with a rather strong signal for C_4_ plants, a δ^13^C value of −17.8, belongs to the gladiator group (EPH-DAM 187/93 rFEM 6) (Fig. 3). Another gladiator (EPH-DAM 248/93 rFEM 6) and one female (EPH-DAM 72/93 rFEM-1) show at least hints for a mixed signal of C_3_ and C_4_ plants. This individual is also extraordinary as she was the only female to be found in the gladiator cemetery. This leads to two hypotheses: 1) these individuals had a different diet than the others or 2) they came from other geographical regions with a more C_4_ plant-based diet and migrated to Ephesus. The second one is more probable because the extensive cultivation of millet is only evidenced since the Early Byzantine Period, ca. AD450, for this region [33]. Comparable δ^13^C data from Roman North Africa (Tunisia) with a mean of −17.7‰ were published by [29]. However, their combined δ^13^C and δ^15^N values suggest a significant amount of dietary protein intake from marine resources.

It is hard to determine how long these individuals from other geographical regions had already lived in Ephesus. Due to collagen turnover in adult bones, the data reflect a mixed value from the final years or even decades of life. Female collagen turnover rates are on average 3–4% per year and reduce with increasing age, whereas for males they are only 1.5–3% per year [43]. Human bone collagen of e.g. femora reflects an individual's nutrition over a much longer time period than 10 years [43]. Most individuals from Ephesus were young adults between 20 and 30 years of age [88]. This also indicates that the three individuals with higher δ^13^C values grew up in other geographical regions and migrated to Ephesus.

### Nitrogen

Compared to other Roman sites, the δ^15^N values of Ephesus are relatively low [16], [21], [23], [28], [29], [30], [33], [114]. The mean value of 9.3‰ is even lower than in Late Imperial Sagalassos (10.1‰, n = 3) but similar to Middle Byzantine Sagalassos (9.1‰, n = 42) in Turkey [33] – even though Sagalassos is far away from the sea and Ephesus is close to the Aegean Sea. Because of its proximity to the ocean and thus good access to seafood, much higher δ^15^N values were expected for Ephesus. Other samples from comparable archaeological Roman sites show a much stronger marine influence (e.g. Isola Sacra in Italy [21], [23] and Leptiminus in Tunisia [29]). The Roman coastal site Velia in southern Italy shows values similar to Ephesus [20]. An ancient cookbook written by *Apicius* which contains a collection of Roman recipes, suggests that seafood was probably consumed in Ephesus. Fish was most likely eaten as fish sauce (*garum*), but also cooked and salted.

The Ephesus δ^15^N values also indicate a generally minor consumption of animal proteins, like meat and dairy products. But the most probable cause for the depletion of ^15^N in Ephesus could have been a frequent consumption of legumes. [34] detected signs of human consumption of pulses in Neolithic Turkey. Archaeo-botanical analyses in Sagalassos, Turkey, show that peas and lentils were extensively cultivated since the Early-Middle Imperial Roman time from 25BC until 300AD [33] and even more in the Late Imperial Roman period.

Legumes generally have very low δ^15^N values due to their molecular nitrogen fixation by symbiotic bacteria which in turn might be reflected in the consumer's collagen. Presumably, the regular consumption of large amounts of pulses lowers human δ^15^N values considerably. Legumes are also rich in protein (up to 25%) and the availability of dietary protein influences the supply of essential amino acids. Wheat for example is short of lysine while beans are short of methionine. A combination of wheat and beans or of cereals and pulses can lead to a protein supply resulting in an availability of up to 90% [34].

Two individuals have much depleted δ^15^N values. One belongs to the gladiator group (EPH-DAM 128/93 rFEM 5) and the second is female (EPH-DAM 106/93 PAR-2). Both relatively low values could be the result of a regular consumption of legumes. Galen reported in his ancient text *De alimentorum facultatibus* that beans (*vicia faba*) were an important nutrition component for gladiators while they explicitly did not get much meat [115]. In contrast to this, the two individuals with the highest values are from the gladiator group, too (EPH-DAM 146/93 rFEM 1 and EPH-DAM 76/93 rFEM 2). That indicates a regular consumption of animal proteins and a lower intake of legumes. Within the gladiator group, the isotope data extend over more than one trophic level for δ^15^N but also for δ^13^C. This leads to the conclusion that the individuals from the gladiator cemetery were a very heterogeneous group who consumed different kind of foods.

### Sulphur

There are only very few comparable sulphur isotope values from Roman sites. [58] investigated humans from a Bronze Age mass burial near Greek Thebes which showed a higher mean for δ^34^S (13.3‰) than the Ephesus individuals (7.4‰). [58] postulates that marine food was rare and that the Bronze Age diet at Thebes mainly derived from terrestrial resources. This could indicate that the individuals from Ephesus also did not consume much seafish or seafood even though the city was close to the Aegean Sea. There are no signs for a high sea-spray effect on the soil of Ephesus either [54], [55].

**Figure 4:**
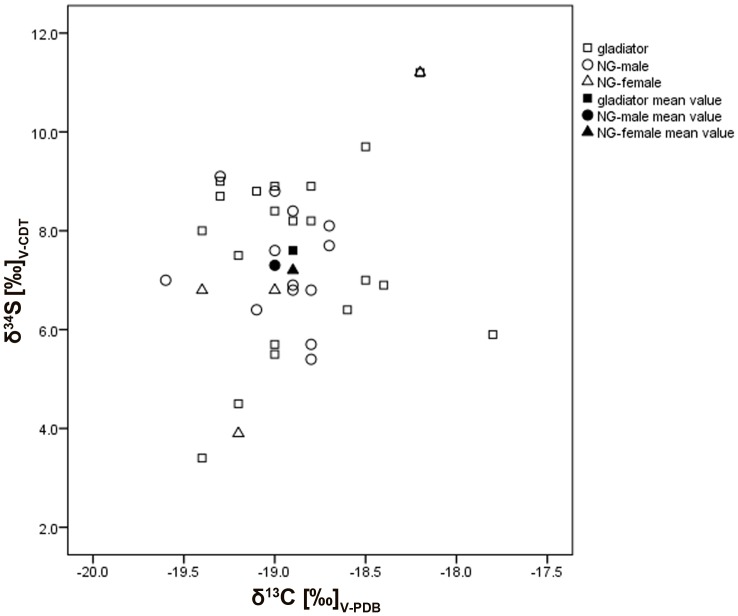
Stable δ^13^C and δ^34^S ratios. Comparison between different groups (gladiators, NG-males, and NG-females).

The female from the gladiator cemetery (EPH-DAM 72/93 rFEM-1) and one gladiator (EPH-DAM 248/93 rFEM 6) show extraordinary δ^34^S values of more than 10‰ (Fig. 4), another gladiator shows δ^34^S values close to 10‰ (9.7‰, EPH-DAM 187/93 r FEM 2). All other individuals have lower sulphur ratios which mean that they can basically be assigned to terrestrial ecosystems. Both individuals with values higher than 10‰ had probably migrated from another geographical region and/or they consumed more fish and seafood than the others [58]. Surprisingly both individuals also show different δ^13^C values. They are discussed above as potential immigrants to Ephesus.

### Trace elements

#### Possible diagenetic alterations

Bio-apatite is extraordinarily chemically reactive because of the living human organism's requirement for the quick regulation of critical elements in the blood [59]. Its good reactivity originates from its high porosity, and the amorphous nature of bio-apatite causes a faster turnover compared to the organic compartment of the bone during life. However, its reactivity does not cease immediately after death, thus making it suspicious for postmortem contaminations. These features of the bone make it susceptible to two main manners of postmortem contamination. Firstly, its high porosity fosters physical infiltration by the soil itself and physical salt deposit from soil solutions. Secondly, the amorphous nature of bio-apatite compared to high crystalline hydroxyl-apatite increases its solubility in wet environments. Additionally, various studies have shown that for all interesting elements contamination can occur in both directions either through enrichment from soil or from depletion to the soil [80]. These processes are unfortunately not unidirectional, furthermore dissolution, permineralization, groundwater replacement, and ion exchange can occur at the same site depending on (ground-)water availability. For this reason, straightforward evaluations of the contamination cannot be made [116]. In general, it seems that virtually all bones buried in soil are affected by some contamination, and so far it does not seem possible to reliably quantify these contaminations [80]. Nevertheless, studies on bone contaminations in various environments prompted tests to find out if massive contamination by the soil has occurred or not [59], [80]. Therefore, possible alteration of Sr/Ca values due to diagenetic changes must be considered in every trace element study.

All investigated human bones and soils were excavated at the foot of the same hill (Panyirdağ) and can be dated within a range of 300 years. We can assume a similar climatic and topographic environment resulting in comparable impact of temperature, rainfall, and ground water conditions for all of the specimens.

All bone samples appeared similar macroscopically with a good status of preservation. Only minor differences of the superficial coloring of the bones were recognized. The same was evident for the hardness of the bones, which could be observed during the homogenization of the bone samples in an agate mortar. On average, about a 15±3% loss of weight during 500°C ashing in the course of sample preparation was detected. This indicates that organic material, believed to be protective for the bone mineral fraction [59], [80], was still present in all of the bone samples.

No quartz residues were found in any of the digestions of bone samples, and measurable concentrations of La in the drill waste bone samples were absent, although La was available (approx. 50 µg/g) in all four investigated soils (Table 2). Presence of quartz and La that are not present in the living bone [117] would be possible predictors for diagenesis in bone due to infiltration of the surrounding soil [59].

However, the prediction of mineral quality is not as simple and straightforward as it is for soil infiltration. Some authors believe that the integrity of the remaining bio-apatite can be checked with the ratio of Ca/P that was found to be about 2.12–2.35 for fresh and archaeological, unaltered bone [102], [104], [118]. Archaeological bones with Ca/P ratios within this range should indicate that no major degradation due to octocalcium phosphate, brushite, whitlockite or significant contaminations with calcium carbonate have occurred. Therefore, a good state of preservation [59] can be assumed. In our study, all investigated bones had Ca/P ratios ranging from 2.16 to 2.35 and therefore meet up to this criterion.

The investigation of the surrounding soil offers more potential to predict significant diagenetic alterations to the Sr/Ca-ratios of the bone. The macroscopic characteristics of all four investigated soils were similarly of dry grey nature. The pH values fell into a range between pH 7.14 and pH 7.72 for all four soils. For soils with pH above 7.0, early trace elements studies postulated that they are less prone to diagenetic alterations of bone than acidic soils (pH<7.0). Nevertheless, [80] found on bones from alkaline soil that they may act as Sr sponges and are able to accumulate Sr to values even higher than 1000 µg/g (mean  =  879 µg/g; max  =  1488 µg/g), although the values for Sr in the surrounding soil were lower. Such extraordinarily high Sr values were not found in or study. Overall we found a mean Sr concentration of 373.9 µg/g, with individual values ranging from 195.6 µg/g to 724.9 µg/g.

The significantly lower Ca values in the DAM93 soil and therefore the much higher Sr/Ca-ratios in this soil raises concerns whether these conditions caused diagenetic alterations of the bones at this location. But, there are still significant differences in the Sr/Ca-ratios between gladiators (1.26±0.33 µg/mg) and non-gladiators (0.63±0.08 µg/mg) from the DAM93 site buried close to each other. Further, our leaching experiments with bi-distilled H_2_O (simulating rain water) for the different soils have shown that although there were great variations in the Sr/Ca-ratios within the different bones and within the different soils, the H_2_O eluents of all four soils turned out to have similar Sr/Ca-ratios, ranging from 2.84 to 3.36 µg/mg (Table 2). [119] studied the ability of bovine bone meal to act as decontamination sorbent for Sr^90^ contaminated soils. The study showed that untreated as well as heated (400°C) bone meal is indeed able to absorb small amounts (about 4 µg/g) of Sr when soaked in aqueous solution with Sr concentrations of as high as 526 µg/L. This happens for a wide range of pH (4 to 11) with more or less the same absorption rate. Additionally, a competition between Ca- and Sr-ions for absorption can be observed when an exceeding concentration of Ca is added to the solutions. Ca ions are clearly preferred to bind at the bio-apatite because of their higher binding stability. These findings indicate for our study that absorption of Sr in the buried bones might have been negligible, because Ca was available in 300 times excess in all four soil eluents.

In theory, sufficiently preserved collagen should protect the metastable calcium phosphate which causes the amorphous nature of the bio-apatite. If the organic (collagen) is lost through burning, the crystallinity of the apatite increases and therefore the solubility of the apatite decreases. This makes the bone less prone to contaminations through ion exchanges [59]. In our study, we found that the cremated samples from the Pithoi showed Sr/Ca-ratios of 0.58 µg/mg respectively 0.61 µg/mg comparable to the uncremated bones. This can be taken as a sign that only minor changes to the original Sr/Ca-ratios have occurred, especially if taking into account that the initially intact and sealed ceramic vessels of the Pithoi prevented the cremated bones from direct groundwater contact at least for a certain time.

Finally, and most importantly, the average Sr/Ca-ratio (0.63±0.08 µg/mg) of the two individuals buried in directly adjoining soil (EPH-DAM 106/93 FEM and EPH-DAM 191/93 rHUM) and the female skeleton buried within the gladiator cemetery (EPH-DAM 72/93 rFEM-1) did not differ significantly from the non-gladiator individuals: 0.68±0.09 µg/mg (asymptotic p = 0.392). Additionally, the Sr values of both gladiators (459.9±125.0 µg/g) and non-gladiators (244.8±32.0 µg/g) are within the physiological ranges reported for modern humans. [120] found a mean of 234 µg/g Sr in 227 humans from around the world ranging from values below 100 µg/g to values as high as 772 µg/g. A later study by [121] from worldwide sampling of 190 individuals revealed Sr values ranging from 100 to 430 µg/g.

Because of the experience many colleagues made during their trace element studies, we assume that diagenetic alterations of the bones must have occurred to some extent. However, we believe that in our study these alterations were not substantial enough to change the Sr signals of the bones which therefore originate from real physiological differences these individuals experienced during their life time.

#### Interpretation of Sr/Ca-ratios

**Figure 5:**
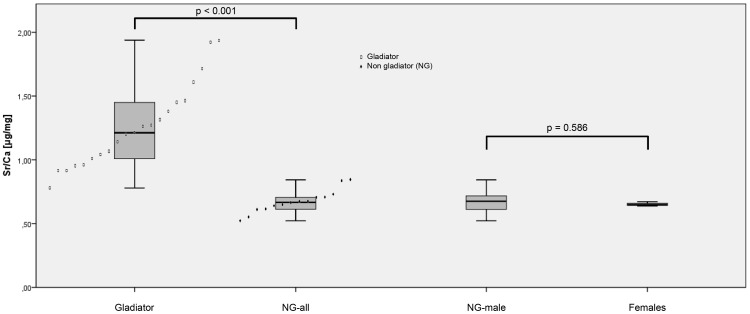
Sr/Ca ratios. Individual values and comparison between different groups (gladiators, NG-all, NG-males, and females).

There is a highly significant difference between the mean Sr/Ca-ratio of the gladiator group and the group of contemporary Romans (Fig. 5). The Sr/Ca-ratio for the gladiators is almost twice as high as for contemporary Romans. The Sr/Ca values of only two contemporary males (EPH-DAM 143/94 lFEM-2 and EPH-DAM 163/94 lHUM 5) showed higher values than the gladiators with the lowest Sr/Ca-ratio (EPH-DAM 155/93 rFEM 4). No individual from the contemporary group reached the second lowest Sr/Ca-ratio in the gladiators group. Using the value for Sr/Ca alone as discriminant allows a certain distinction between gladiators and non-gladiators.

[122] observed Sr concentrations (299.0±41.1 ppm) in a late Roman population from Southern Germany (n = 70) which are similar to our ordinary Roman group (244.8±32.0 ppm) but much lower than the Sr concentrations of the gladiator group (459.9±125.0 ppm). Comparable differences between high and low Sr/Ca-ratio values in groups with a distinct social status are reported for the Early Medieval Weingarten in Southern Germany [123]. In this study, a group of 90 individuals associated to a low social status showed an average Sr/C-ratio of 1.44±0.16 µg/mg. A second group of 69 individuals associated to a high social status showed an average Sr/Ca-ratio of 0.90±0.19 µg/mg. [123] suggest, supported by stable isotopes data, that the diet of the group with the high Sr/Ca-ratio consisted of both low-Ca vegetable food and green plants providing high Ca and Sr levels. The group with the low Sr/Ca was assumed to have had a clearly Ca-enriched diet including a considerable amount of milk and dairy products that supply the organism with highly bio-purified Ca [123].

In this study, the isotopic data suggest a mixed diet with a high share of vegetables. However, this cannot fully explain the great difference in Sr/Ca-ratios. Other possible reasons for the high Sr/Ca values of gladiators must be discussed. The stable isotope data means do not differ significantly between gladiators and contemporary females and males, suggesting that there were no major differences in their overall diet. However, the significantly higher Sr/Ca-ratio for the gladiators is a very strong signal of the main Ca-supplier in their diet [79]. A Ca-source not detectable in the isotopic signature must be responsible for such significant differences in Sr/Ca-ratios between the gladiators and non-gladiators.

In a study on the non-linearity of bone Sr/Ca and diet, [79] describe *bivilviki*, a traditional Hopi food made from corn to which ash of the salt bush plant is added. They conclude that the Sr/Ca-ratio of *bivilviki* is responsible for the consumer's bone Sr/Ca, even if it was just a minor part of the diet. Plant ash is known as a culinary spice and also as a medical remedy in many cultures. It is frequently mentioned as *pyxis* in Roman texts. In his *Naturalis historia*, Pliny the Elder describes a beverage made of stove ashes that played a role in the life of gladiators. This ash beverage was served after fights and maybe also after training to remedy body pain [124]. It is a possible explanation for the high Sr/Ca values in the gladiator bones.

The strong Sr/Ca signal in the gladiator bone mineral indicates an accelerated Ca metabolism. According to historic sources, a gladiator spent several years in the *ludus*. The first year served for initial training followed by at least three years as active fighter [8], [9]. The consumption of the ash beverage during this time period would suggest that our gladiators may comprise fighters who lost their life in the first fight and others who lost their lives after several years. That means a substitution for the novices of approximately one year and several years of substitution for the more experienced fighters. A strong gradient or a high variation of Sr/Ca-ratios within our gladiator samples could reflect these different time spans (Fig. 5).

The high Sr/Ca ratios of the gladiators compared to the contemporary group are nevertheless hard to obtain by a regular daily Ca bone exchange rate. This would lead to a complete turnover of the 1000–1500 g total body Ca after more than five to eight years if each Ca atom is subsequently replaced. On the one hand, Sr tends to substitute especially in newly formed bone in the course of a high Sr intake [125], [126]. This is also supported by several studies (animals and humans) on Sr incorporation in bone during Sr-ranelate treatment of osteoporosis [127], [128], [129] which found a clear link between incorporated Sr and serum levels. Additionally, recent studies [128], [129] found a non-linear increase of Sr levels with Ca-content, and therefore postulate that the accumulation process of Sr ions in the apatite crystals may be still ongoing even after the crystals had stopped growing by further ion substitution. On the other hand, there is evidence of increased bone mineral density and bone formation for bone sites under high mechanical stress. This was shown for example when triathletes were compared to athletes with less external loading on the skeleton like swimmers and cyclists [130]. Elloumi et al. [131] observed a significantly accelerated formation of bone mineral content and increased bone formation marker osteocalcin in a similar study of modern rugby players. Especially the adult and older individuals showed enhanced bone mass and increased bone turnover in their long bones due to intense training. If gladiators also underwent an intense and constant training that included running and jumping with heavy protection gear – hence causing high mechanical stress on their weight-bearing bones – then an accelerated Ca and Sr metabolism can be assumed.

Feeding experiments on laboratory rats demonstrated how rapidly Sr supplementation evolves as a strong signal on the bone Sr content [132]: After three months on a high Sr diet (16.58 µg/mg), male rats showed an average bone Sr/Ca-ratio of 5.67 µg/mg whereas in the control group with low Sr diet (0.55 µg/mg) the average Sr/Ca-ratio was 0.13 µg/mg. After five weeks, the bone Sr/Ca-ratios had increased by thirty times.

Feeding studies by Pagano et al. [133] investigating the strontium uptake of young pigs to enhance their bone strength might reflect even better the processes in humans. In their second experiment they fed pigs for 5 weeks with a diet with and without substituting50 mg/kg Sr (using SrCO_3_) to the diet. After five weeks, Sr/Ca-ratios in the compacta of the femora of the pigs were 0.27 µg/mg in the control group and as high as 1.40 µg/mg in the substituted group, thus evidencing a more than fivefold increase of the Sr/Ca-ratio. Nevertheless, caution is required when applying these findings to the interpretation of our data, because growing animals were used in the study whereas in our study only adult individuals were included. Therefore, a higher turnover, respectively developing rate, can be assumed for the animal study.

Even though the real amount of contamination cannot be reconstructed with certainty, there are strong signs for a real physiological difference in the Sr/Ca-ratios of gladiators and non-gladiators. Therefore, we cautiously suggest that there must have been differences in the diet of these two groups. In particular, there must have been a food component for the gladiator group which served as additional Ca supplier. This component did not leave any signature in the stable isotopes ratios, but did leave a significant increase of the Sr/Ca-ratios. One possible explanation would be a plant ash drink which is mentioned in a few historical texts. Assuming that power athletes need about 2000 mg of Ca substitution per day and if even only half of this came from ash drink, then this could explain the Sr/Ca values. It would mean that theoretically even 1 g of plant ash per day would already be sufficient to explain the high Sr/Ca-ratios. Nevertheless, we cannot exclude some other mineral uptake of an unknown Ca supplier, such as dairy products which did not show significant signatures in stable isotope ratios.

Taking all these observations into consideration (enhanced bone mineral content formation due to intense physical training, fast Sr/Ca changes due to Sr enriched food supplements, strong relation of Sr/Ca of the major Ca supplier in the diet), the use of the ash beverage seems to be a valid explanation. Some wood types that were common during the Roman period in the Mediterranean are possible sources for the ash Sr/Ca-ratios [134]. Cedar, pine, and oak show Sr/Ca-ratios between 1.42 µg/mg and 2.10 µg/mg; a particularly high ratio of 5.34 µg/mg was found in the poplar, which is still a common tree in the region around Ephesus.

### Limitation of the study

Analysis of animal bones provides important information on the general status of the whole population. Therefore, human-faunal trophic level should be taken into consideration. Variations in the stable isotope and trace element data in comparison to other Roman burial sites could also be the result of different management of animals or environmental conditions. Stable isotope and trace element data of the animal bones from Ephesus are subject to ongoing research and will be presented in future work.

## Conclusions

The stable isotope values show that all individuals consumed C_3_ plants, such as wheat and barley as staple food. A few individuals also show signs for consumption of C_4_ plants. The different groups' (gladiators, NG-males, females, infants) stable isotope data do not differ significantly. The δ^15^N values of Ephesus are relatively low compared to other sites from Roman times.

There are two data outliers: one female from the gladiator burial site and one gladiator. These two individuals probably migrated from another geographical region or had a different diet. The data presented are among the few isotope palaeodiet studies based on a large sample of human bone material from Roman populations of Turkey. Because we did not find any hints for substantial diagenetic alterations, we believe that the significant differences in Sr-concentrations have predominantly physiological reasons. If we accept real physiological differences, arising from different diet habits, then the high Sr/Ca-ratio for gladiators seems to be associated to a specific alimentation during their years spent at the fighter's school.
